# Bone marrow mesenchymal stem cells improve bone erosion in collagen-induced arthritis by inhibiting osteoclasia-related factors and differentiating into chondrocytes

**DOI:** 10.1186/s13287-020-01684-w

**Published:** 2020-05-07

**Authors:** Jinfang Gao, Gailian Zhang, Ke Xu, Dan Ma, Limin Ren, Jingjing Fan, Jianwen Hou, Jian Han, Liyun Zhang

**Affiliations:** 1 Department of Rheumatology, Shanxi Bethune Hospital, Taiyuan, 030032 Shanxi China; 2 Department of Rheumatology, Bethune Hospital Affiliated to Shanxi Medical University, Taiyuan, 030032 Shanxi China

**Keywords:** Bone marrow-derived mesenchymal stem cells, Rheumatoid arthritis, Bone destruction, Green fluorescent protein, Tissue repair, RANKL/OPG, Chemokines

## Abstract

**Background:**

Rheumatoid arthritis (RA) is characterized by joint inflammation and damage to the cartilage and bone in collagen-induced arthritis (CIA). Mesenchymal stem cells (MSCs) can improve articular symptoms and reduce bone erosion in CIA rats; however, the underlying mechanism remains unknown. This study aimed to investigate the mechanism underlying MSC-induced improvement of bone destruction in CIA.

**Methods:**

Wistar rats were divided into a normal group, CIA control group, MTX intervention group, and BMSC intervention group, each comprising 8 rats. Serum RANKL, OPG, and CXCL10 levels of all groups were determined via flow cytometry after 42 days of interventions. RANKL, OPG, TRAF6, CXCL10, and CXCR3 were detected on the synovial membrane via immunohistochemistry, and their relative mRNA levels were determined via RT-PCR analysis. BMSCs were labeled with GFP and administered to CIA rats via the tail vein. At different time points, the distribution of implanted GFP-MSCs in synovial tissues was observed using a fluorescence microscope, and the potential of GFP-MSCs to differentiate into chondrocytes was assessed via immunofluorescence analysis.

**Results:**

BMSC transplantation improved joint inflammation and inhibited bone destruction in CIA rats. BMSCs inhibited the expression of serum CXCL10 and CXCL10 and CXCR3 expression at the synovial membrane. Moreover, protein and mRNA expression analyses revealed that BMSCs potentially regulated RANKL/OPG expression levels in the serum and synovial tissue. Upon implantation into CIA rats, GFP-MSCs were traced in the joints. GFP-positive cells were observed in the cartilage tissue from day 11 and until 42 days after transplantation. Anti-type II collagen/GFP double-positive cells were observed in the articular cartilage (especially damaged cartilage) upon immunofluorescence staining of anti-type II collagen.

**Conclusions:**

BMSCs improve bone destruction in CIA by inhibiting the CXCL10/CXCR3 chemotactic axis, regulating the RANKL/OPG ratio, and directly differentiating into chondrocytes.

## Introduction

Rheumatoid arthritis (RA) is characterized by symmetric polyarthritis. Synovial inflammatory infiltration and the interaction between immune cells and synovial fibroblasts ultimately result in cartilage and bone erosion. Traditional RA drugs primarily include non-steroidal anti-inflammatory drugs and slow-acting anti-rheumatic drugs (SAARDs), which often cause gastrointestinal side effects, kidney damage, bone marrow suppression, and psychological disorders. Recent advancements in studies on RA have elucidated the role of inflammation, autoimmunity, cytokine networks, and various cellular functions in RA occurrence and pathogenesis. Various biological agents that block cytokines, including TNF-α, IL-1, and IL-6, and target B cells and osteoclasts have been reported, having benefited numerous refractory RA patients. However, such drugs exclusively target a certain step in RA pathogenesis and are costly. They can induce reactions at the site of injection, tumors, and infectious diseases including tuberculosis, characterized by easy relapse after drug withdrawal. Moreover, approximately 30% of patients do not respond to this treatment, and RA patients with clinical remission display progressive joint erosion upon imaging examination [1, 2]. Therefore, upon joint damage in RA, joint erosion cannot be effectively repaired through pharmacotherapeutic intervention, thereby serving as a risk factor for progressive joint damage, secondary osteoarthritis, and joint dysfunction.

Advancements in cell, tissue, and genetic engineering have rendered stem cell therapy suitable for RA. Mesenchymal stem cells (MSCs) can be easily isolated and amplified, are multipotent, especially differentiating into chondrocytes, and contribute to immunoregulation and anti-inflammatory functions. Hence, they are ideal for repairing joint injuries in RA patients. Numerous studies have reported that MSCs have immunomodulatory effects in RA; however, no studies have reported the fate of MSCs implanted in RA patients or animal models of RA. The mechanisms underlying the migration of MSCs and their homing to joint tissue and the inhibition of bone destruction in rats upon external administration of MSCs remain unknown.

In this study, a rat model of CIA for RA was administered GFP-labeled MSCs via the tail vein, and the migration and homing of bone marrow MSCs (BMSCs) were traced and the mechanism underlying the inhibition of bone destruction in damaged joints was investigated.

## Materials and methods

### Isolation, culturing, and identification of rat BMSCs

#### Isolation and culture of rat BMSCs

This study was approved by the ethical committee of Shanxi Bethune Hospital (Shanxi Dayi Hospital). Rat BMSCs were isolated and cultured as previously described [3, 4]. Briefly, rat BMSCs were harvested from the bone marrow of posterior tibias and femurs of 2-week-old Wistar rats. After centrifugation at 200×*g* for 10 min, cells were resuspended in α-MEM (Gibco, Grand Island, NY, USA) supplemented with 10% fetal bovine serum (FBS; Gibco) and cultured at a density of 4 × 10^4^/cm^2^ at 37 °C in moist air and 5% CO_2_. After three passages, cells were used for experiments.

#### Identification of rat BMSCs

##### Identification of the surface antigen phenotype of BMSCs

Suspensions of BMSCs of the P3 generation were prepared (1 × 10^6^ cells/mL). Cells were incubated on ice for 30 min with anti-rat antibodies (BioLegend, USA) conjugated with fluorescein isothiocyanate (FITC) or phycoerythrin (PE). Markers of BMSCs (CD 44-PE, CD105-FITC, and CD29-PE), hematopoietic cells (CD45-FITC), and endothelial cells (CD31-PC5 and CD34-PC5) were evaluated and analyzed respectively via flow cytometry.

##### Identification of multipotent differentiating BMSCs

To assess multipotential differentiation, the cells were digested with 0.25% trypsin and transplanted into a 6-well plate and then cultured with osteogenic, adipogenic, or chondrogenic induction medium for 21 days.

The osteogenic induction medium comprised L-DMEM supplemented with 10% FBS, 100 nmol/L dexamethasone, 10 mmol/L sodiumβ-glycerophosphate, and 0.05 mmol/L l-ascorbic acid 2-phosphate and was replaced every 3 days. Alizarin Red staining was performed to analyze the osteogenic potential.

To analyze adipogenic differentiation, cells were incubated in H-DMEM medium supplemented with 1 mmol/L dexamethasone, 0.2 mmol/L indomethacin, 10 mg/mL insulin, 0.5 mmol/L 3-isobutyl-1-methylxanthine, and 10% FBS for 21 days. The adipogenic induction medium was replaced every 3 days. Oil red O staining was performed to analyze the adipogenic potential.

To analyze chondrogenic differentiation, cells were cultured in 2 mL of induction medium, supplemented with 10 ng/mL rhTGF-β1, 1 mmol/L sodium pyruvate, 10^− 7^ mmol/L dexamethasone, 6.25 g/mL bovine insulin, 0.5 μL/mL Vc, and 5% fetal bovine serum H-DMEM. The medium was changed every 3 days, and the cells were observed daily using an inverted microscope. At 14 days after induction, the slides were removed and the cells were stained with methylaniline blue (Sigma, Germany), and immunohistochemical staining was performed for type II collagen (Abcam, Cambridge, MA, USA, cat. no. ab34712) with SABC (ready-to-use SABC-ap (rabbit IgG) kit, BOSTER, Wuhan, China).

### Experimental animals

Wistar rats (female, 6–8 weeks old, weighing 140–150 g, *n* = 40) were purchased from Beijing Weitong Lihua Laboratory Animal Technology and housed at the Shanxi Medical University Animal Center at a controlled temperature of 20 °C, a relative humidity of 50%, and a photoperiod of 8 h. All experiments were conducted in accordance with the guidelines of the animal protection and use committee of Shanxi Medical University.

The rats were segregated into the normal group (*n* = 8) and the CIA model group (*n* = 32). Rat models of CIA were generated through immunization with type II collagen as previously described [5]. Briefly, upon anesthesia with 5% chloral hydrate, the rats were injected with mixed emulsions containing 0.1 mL of complete Fresno adjuvant and 400 μg of bovine type II collagen (Sigma-Aldrich, St. Louis, MO, USA) on the dorsa, the root of the tail, and the hind paws. The rats in the blank group were similarly administered physiological saline. After 2 weeks, CIA model rats were intraperitoneally administered a 0.2-mL emulsion containing 1 mg/mL bovine type II collagen for booster immunization. The blank group was administered the same amount of physiological saline.

The thirty-two CIA rats were randomly assigned to four groups: CIA control group (CIA group, *n* = 8), methotrexate intervention group (MTX group, *n* = 8), BMSC intervention group (BMSC group, *n* = 8), and GFP-MSC intervention group (GFP-MSC group, *n* = 8). In the BMSC intervention group, 1 × 10^7^ BMSCs were transplanted into the tail vein of each rat once after booster immunization. The normal group and CIA model group, as the control groups, were similarly administered an equal volume of PBS at the same time point. The MTX group was intraperitoneally administered 0.9 mg/kg body weight MTX. Each group was housed separately throughout the study.

### Analysis of the effect of BMSCs on CIA model rats

To grossly evaluate joint inflammation and osteochondral injury in CIA model rats in the CIA group, BMSC group, and methotrexate group, assessment of arthritis index (AI), imaging, and pathological analysis were performed. The rats of the GFP-MSC group were used for the assessment of cell migration, colonization, and differentiation in CIA model rats.

Joint inflammation was assessed via AI weekly from primary immunization to 5 weeks after intervention at eight time points. AI was graded on a scale of 0–4 [6]: 0 = no evidence of hyperemia and/or inflammation; 1 = hyperemia with minor or no paw swelling; 2 = swelling confined predominantly to the ankle region, with modest hyperemia; 3 = increased paw swelling and hyperemia of the ankle and metatarsal regions; and 4 = maximal paw swelling and hyperemia involving the ankle, metatarsal, and tarsal regions. The scores of four hind paws were combined to obtain the AI for each rat, with a maximum possible score of 16.

X-ray radiographic (Philips Corporation, NY, USA) assessment and micro-computed tomographic (micro-CT) assessment (SCANCO Medical AG, Zurich, Switzerland) of joint destruction were conducted on day 6 after intervention independently by two experienced physicians in a blinded manner. Using the CTAn software to analyze micro-CT images, we obtained bone structure parameters such as bone volume/tissue volume (BV/TV), trabecular number (Tb. N), trabecular thickness (Tb. Th), and trabecular separation (Tb. Sp). For histological analysis, rats were euthanized via cervical dislocation during the 6th week. Joint tissues were randomly harvested, embedded in paraffin, sectioned, and stained with hematoxylin and eosin (H&E).

### Effects of BMSCs on RANKL/OPG/TRAF6 signaling and the CXCL10/CXCR3 chemokine axis in CIA model rats

#### Serum RANKL, OPG, and CXCL10 levels

Rats in the normal group, CIA group, MTX group, and BMSC group were anesthetized with 10% chloral hydrate at a dose of 4.5 mL/kg after 6 weeks of intervention. The blood was sampled with a capillary tube from the eye frame. After centrifugation, the supernatant was obtained and stored at − 70 °C. Levels of the bone metabolism-related factors RANKL, OPG, and chemokine CXCL10 in serum were detected via flow cytometry. Serum cytokine concentrations were measured with a commercially available kit (Aimplex® Rat Custom 9-plex kit, Beijing Kuangbo Biotechnology Co., Beijing, China) in accordance with the manufacturer’s instructions, using a flow cytometer (BD Biosciences, Franklin Lakes, NJ, USA). CellQuest software (BD Biosciences) and the Windows Multiple Document Interface flow cytometry application were used for data analysis.

#### RANKL/OPG/TRAF6 signaling and the CXCL10/CXCR3 chemokine axis in synovial tissues

##### Sample preparation

After blood sampling, rats were euthanized via cervical dislocation, and the synovial tissue was promptly resected for further analysis. Fresh tissue was divided into two parts: one part was fixed in 4% paraformaldehyde overnight and embedded in paraffin for immunohistochemical analysis; the other part was stored in a liquid nitrogen container at − 200 °C for reverse transcriptase PCR (RT-PCR) analysis.

##### Immunohistochemical analysis

The expression of RANKL, OPG, TRAF6, CXCL10, and CXCR3 in synovial tissues was analyzed through immunohistochemical analysis. The paraffin-embedded synovial tissue was cut into 5-μm sections using a microtome (Leica, Wetzlar, Germany) and dried at 30 °C for over 4 h. After deparaffinization, the sections were incubated in 3% H_2_O_2_ at room temperature for 10 min to block endogenous peroxidase. After antigen retrieval, the sections were incubated overnight at 4 °C and then for 40 min at room temperature with anti-RANKL polyclonal antibody (Abcam, Cambridge, MA, USA; cat. no.ab169966), anti-OPG polyclonal antibody (Abcam, Cambridge, MA, USA; cat. no. ab203061), anti-TRAF6 monoclonal antibody (Abcam, Cambridge, MA, USA; cat. no. ab40675), or anti-CXCR3 monoclonal antibody (Boshide Biology Company, Shenzhen, China). The sections were washed with phosphate-buffered saline (PBS) and then incubated with polymer helper (Boster Biological Technology, China) for 20 min and anti-rabbit IgG (Zsbio, Beijing, China, no. 31466) for 30 min at 37 °C. The sections were washed with PBS and incubated with polymer helper for 20 min and anti-rabbit IgG for 30 min at 37 °C. The sections were stained with DAB and observed using an optical microscope. When the cytoplasm stained brown, the reaction was terminated using tap water. The tissue was re-dyed with hematoxylin, dehydrated, clarified, finally sealed with gum, and then imaged using an optical microscope (Olympus BX41, Tokyo, Japan).

##### RT-PCR analysis

The expression of RANKL, OPG, TRAF6, CXCL10, and CXCR3 mRNAs was analyzed through RT-PCR analysis. Total RNA was extracted from the synovial tissue using TRIzol reagent (Invitrogen, Carlsbad, CA, USA), and the lysate was centrifuged at 10,000×*g* and 4 °C for 15 min. The water sample layer was transferred to another new Eppendorf tube, and the RNA was precipitated with ice-cold isopropanol. Spectrophotometric analysis was performed to determine the A_260/280_ ratio to determine the RNA quality, which ranged from 1.8 to 2.0, and the RNA concentration ranged from 1000 to 2000 μg/mL. Glyceraldehyde-3-phosphate dehydrogenase (GAPDH) was used as the internal control to analyze the mRNA expression levels and DNA synthesis for each sample experiment. Optimal PCR primers were designed using the Primer 3.0 software and simultaneously compared with sequences previously uploaded to the GenBank. The primer sequences were as follows: GAPDH—F5′-TGCACCACCAACTGCTTAGC-3′, R5′-GGCATGGACTGTGGTCA TGAG-3′; RANKL—F5′-CATCGGGTTCCCATAAAG-3′, R5′-GAAGCAAATGTTGGCGTA-3′; OPG—F5′-TTGGCTGAGTGTTCTGGT-3′, R5′-TTGGGAAAGTGGTATGCT-3′; TRAF6—F5′-CTGCTTGATGGCTTTACGGG-3′, R5′-ATGGGCACAGCACAGTTTAC-3′; CXCL-10—F5′-TCTCTCCACCTCCCTTTACCC-3′, R5′-CTTGTCCATCACGCTGTAGT-3′; and CXCR3—F5′-ACATCAACTTCTACGCAGGG-3′, R5′-CACACAACAATGCAGGTGAGG-3′. In accordance with the manufacturer’s instructions, Rotor-Gene SYBR Green PCR Kit (Qiagen, Hilden, Germany) was used to perform RT-PCR on a real-time fluorescence quantitative PCR analyzer (Qiagen). Each sample was analyzed in triplicate.

### Assessment of the migration, colonization, and differentiation of GFP-BMSCs in CIA model rats

#### Obtainment of GFP-MSCs

Commercially obtained GFP-MSCs (OriCell F344 rat bone marrow MSCs/GFP, Cyagen Biosciences, CA, USA) were resuscitated, cultured, and subcultured, and then cell morphology was observed using a fluorescence microscope (Olympus B51, Tokyo, Japan). Thereafter, 1 × 10^7^ cells/kg of GFP-MSCs were administered to CIA model rats through the tail vein after booster immunization.

#### Assessment of the migration, colonization, and differentiation of GFP-BMSCs in CIA model rats

After transplantation, 2 rats were euthanized at 3, 11, 30, and 42 days after intravenous GFP-MSC administration, and paraffin-embedded sections of the posterior ankle joints were obtained. The distribution of GFP-MSCs in the articular cartilage was assessed using a fluorescence microscope (OLYMPUS B51, Tokyo, Japan).

After 42 days of implantation, the paraffin-embedded sections of the ankle joints of rats were subjected to immunofluorescence staining, using rabbit anti-rat type II collagen antibody (Abcam, Cambridge, MA, USA, cat. no. ab34712) as the primary antibody and rhodamine (TRITC)-conjugated goat anti-rabbit IgG (H+L) (Zsbio, Beijing, China, no. AS040) as the secondary antibody. The same field was observed using a laser-scanning confocal microscope. The DAPI-stained nuclei were observed upon excitation with blue light, GFP-positive cells (i.e., transplanted GFP-MSCs) were observed upon excitation with blue light, and type II collagen-positive cells were observed upon excitation with red light. The image analysis software (Imstar, French) was used to overlay three differently colored images under the same field to detect GFP/type II collagen double-positive cells.

### Statistical analysis

The data were analyzed using the statistical software SPSS 22.0. The AI of each group at different time points was compared using repeated measures ANOVA. Normally distributed data with homoscedasticity are expressed as mean ± standard deviation, and the comparisons among the groups were carried out using one-way ANOVA followed by the LSD test for post hoc multiple comparisons. Non-normally distributed data are expressed as median (quartile spacing) values, and the comparisons among the groups were performed using the Kruskal-Wallis rank sum test, followed by Nemenyi test for post hoc analysis. *P* values less than 0.05 were considered statistically significant, and for all figures, *P < 0.05, **P < 0.01, and ***P < 0.001.

## Results

### Isolation, culture, and identification of rat BMSCs

**Fig. 1:**
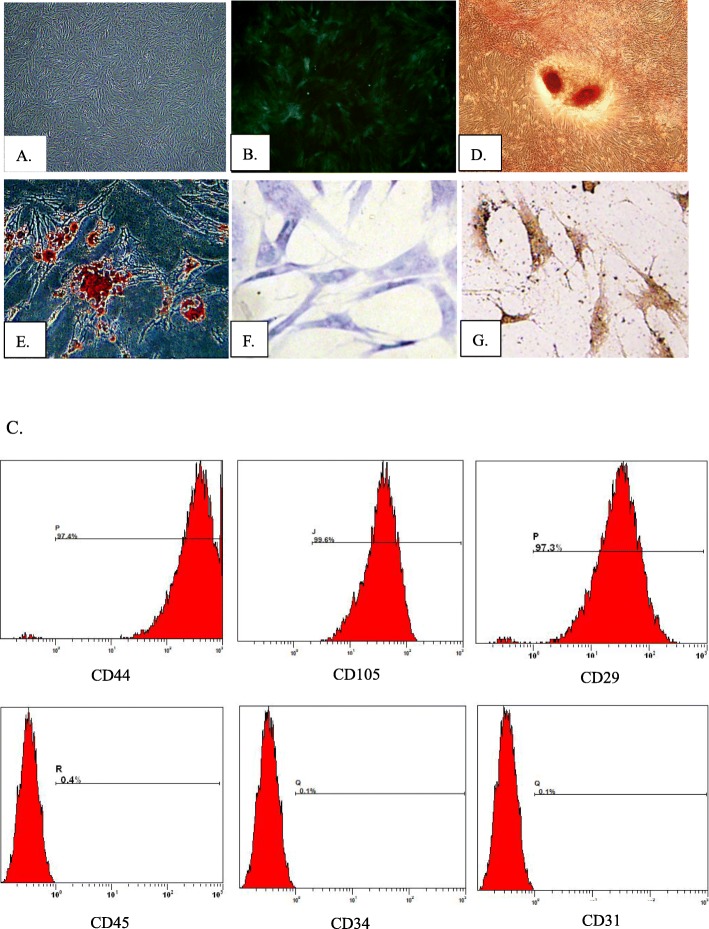
Characteristics of BMSCs and GFP-BMSCs. **a** Cell culture of passage 3 (original magnification × 100). **b** GFP-BMSCs under the fluorescence microscope (original magnification × 100). **c** Flow cytometry results showed that cells were positive for CD44, CD105, and CD29, while negative for CD45, CD34, and CD31. **d** Osteogenic differentiation of BMSCs indicated by Alizarin Red staining (original magnification × 100). **e** Oil red O staining of BMSCs after induction of adipogenic differentiation (original magnification × 100). **f**, **g** After induction of chondrogenic differentiation, toluidine blue staining and collagen type II immunohistochemical staining were positive (original magnification × 200)

Rat BMSCs grew by adhering to the plastic substratum under standard culture conditions and displayed a long spindle-like morphology (Fig. 1a). Flow cytometry analysis of BMSC surface antigen phenotype revealed that CD44, CD105, and CD29 were expressed, while CD45, CD34, and CD31 were not (Fig. 1c). Moreover, upon culturing in an induction medium, BMSCs possessed adipogenic, osteogenic, and chondrogenic differentiation potentials in vitro (Fig. 1d–g). Furthermore, fluorescence microscopic assessment revealed that the morphology of the commercially purchased GFP-MSCs was similar to that of rat BMSCs extracted and cultured in our laboratory, and the cells displayed a green fluorescence signal (Fig. 1b).

### Effects of BMSCs on CIA rats

#### General conditions

**Fig. 2:**
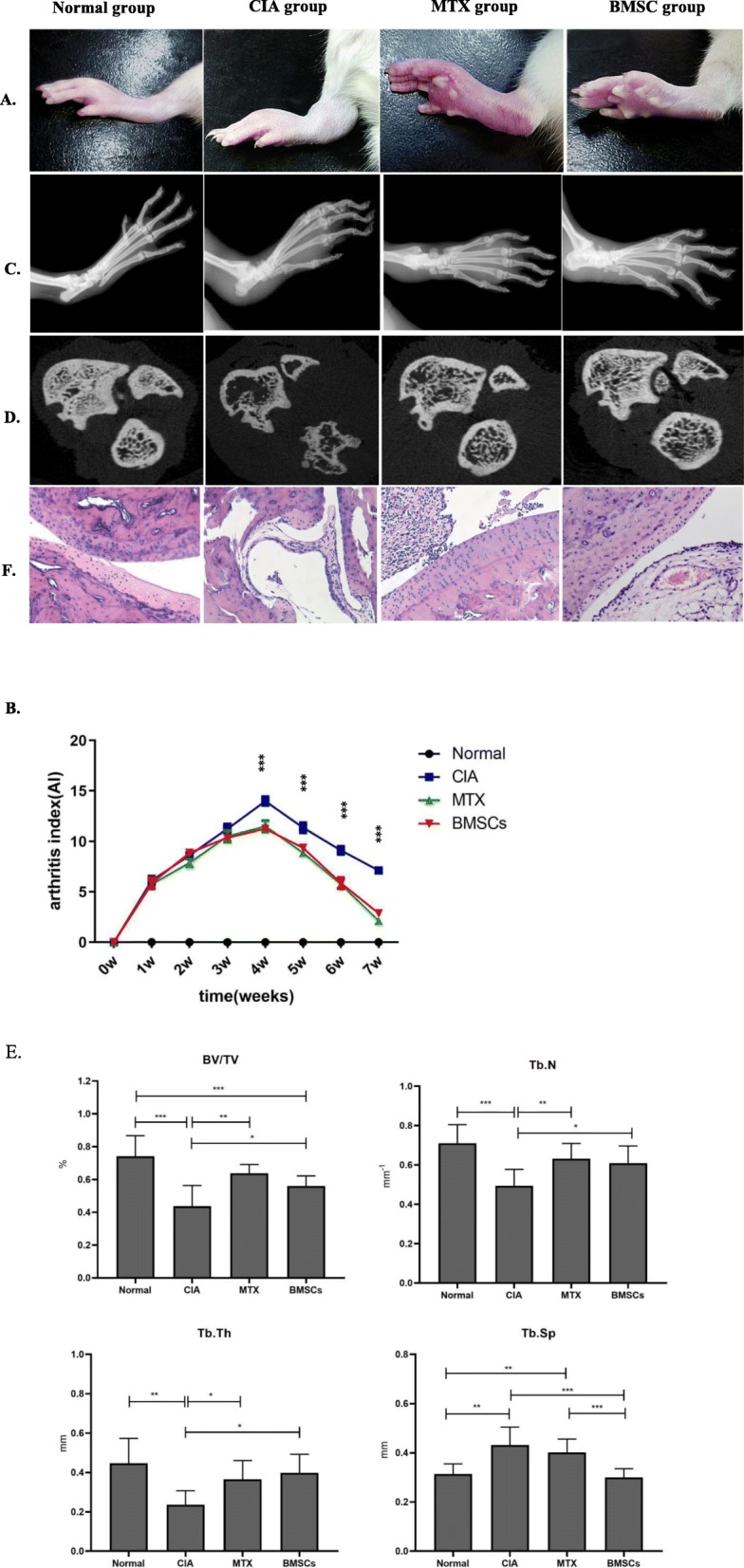
BMSCs alleviated inflammatory responses and prevented joint damage in CIA rats. **a** Swelling of the ankle joints in CIA rats was obvious and relieved after BMSCs or MTX intervention. **b** Compared with the normal group, the arthritis index (AI) value in the CIA model group increased; however, it showed a downward trend from the second week after BMSC and MTX intervention. *P < 0.05; **P < 0.01; ***P < 0.001. **c** X-ray image showed that CIA rats had a narrow ankle and toe joint space, bristle edge, and joint destruction, and joint erosion was reduced after BMSC or MTX intervention. **d** Significant osteoporosis and bone destruction were observed in the ankle joint of CIA rats but improved in the BMSC and MTX intervention groups on micro-computed tomographic imaging. **e** Compared to the CIA group, BMSCs can increase the level of BV/TV, Tb. N, and Tb. Th and reduce the value of Tb. Sp. **f** Joint pathology findings suggested that synovial hyperplasia, inflammatory cell infiltration, and cartilage destruction were observed in the CIA rat group but were attenuated in the BMSC and MTX intervention group (HE, × 20)

After initial immunization of the CIA control group, the luster of rat fur was gradually lost and feeding was reduced; however, the degree of swelling of the ankle joint and toe joint gradually increased. The first inflammatory peak was observed approximately 10 days after the initial immunization. Another inflammatory peak was observed 4 weeks after the initial immunization. No adverse reactions were observed in the BMSC and MTX intervention groups. Furthermore, rats in both intervention groups presented with hypochromia, decreased feeding, and joint swelling, which were significantly lesser than those in the CIA group. No manifestations of joint ankylosis were observed in the intervention group (Fig. 2a).

#### Comparison of the AI in each group

Joint inflammation was assessed by AI weekly from primary immunization to 5 weeks after intervention for eight time points. Except for the normal group, the AI value of the other three groups generally showed an upward trend with the passage of time in the first 5 weeks. No statistical difference was observed in AI among the three groups. In the last 3 weeks, the average AI value of the CIA, MTX, and BMSC groups showed a gradual decline trend. Compared with that of the CIA group, the AI values of the MTX and BMSC groups were significantly decreased (*P* < 0.001); however, the difference between the two groups was not significant (*P* = 1) (Fig. 2b).

#### Joint imaging findings

Regular X-ray images of the hindlimbs of rats were obtained, and no obvious osteoporosis was observed in the joints in the control group; furthermore, the joint space and the bone were intact. Rats in the CIA group may have had osteoporosis, and the joint space was markedly narrowed; furthermore, joint destruction and fusion were observed to a minor extent. Joint damage in the BMSC intervention group was lesser than that in the CIA control group (Fig. 2c). Micro-CT examination indicated that the trabeculae in the blank control group were neatly arranged, with a dense structure and complete bone microstructure. In the CIA group, the proximal femoral cortex was thinner; the trabeculae were sparse, thin, disordered, and fewer; and the intertrabecular distance was increased. Cortical thickness in the BMSC group and MTX group was the same as that in the blank control group, and the number of trabeculae decreased slightly but increased significantly compared with that in the model group (Fig. 2d). The analysis results of bone structural parameters showed that, compared with those in the normal group, BV/TV, Tb. N, and Tb. Th of the CIA group were significantly reduced, while Tb. Sp was increased. The values of BV/TV, Tb. N, and Tb. Th were dramatically higher in both the MTX and MSC groups than in the CIA group. The Tb. Sp of both intervention groups was decreased, but only the MSC group showed a statistical significance. There was no statistical difference in the Tb. Sp value between the MSC and normal control groups (Fig. 2e).

#### Pathological joint manifestations

Hematoxylin and eosin staining of pathological sections in the normal group revealed no abnormal synovial hyperplasia, and the cartilage structure was intact. In the CIA and MTX groups, synovial hyperplasia was obvious, with villar protrusion, inflammatory cell infiltration, and articular cartilage defects. Compared with the CIA group, the BMSC intervention group displayed mild synovial hyperplasia and mild articular cartilage destruction (Fig. 2f).

### Serum RANKL, OPG, and CXCL10 levels

**Fig. 3:**
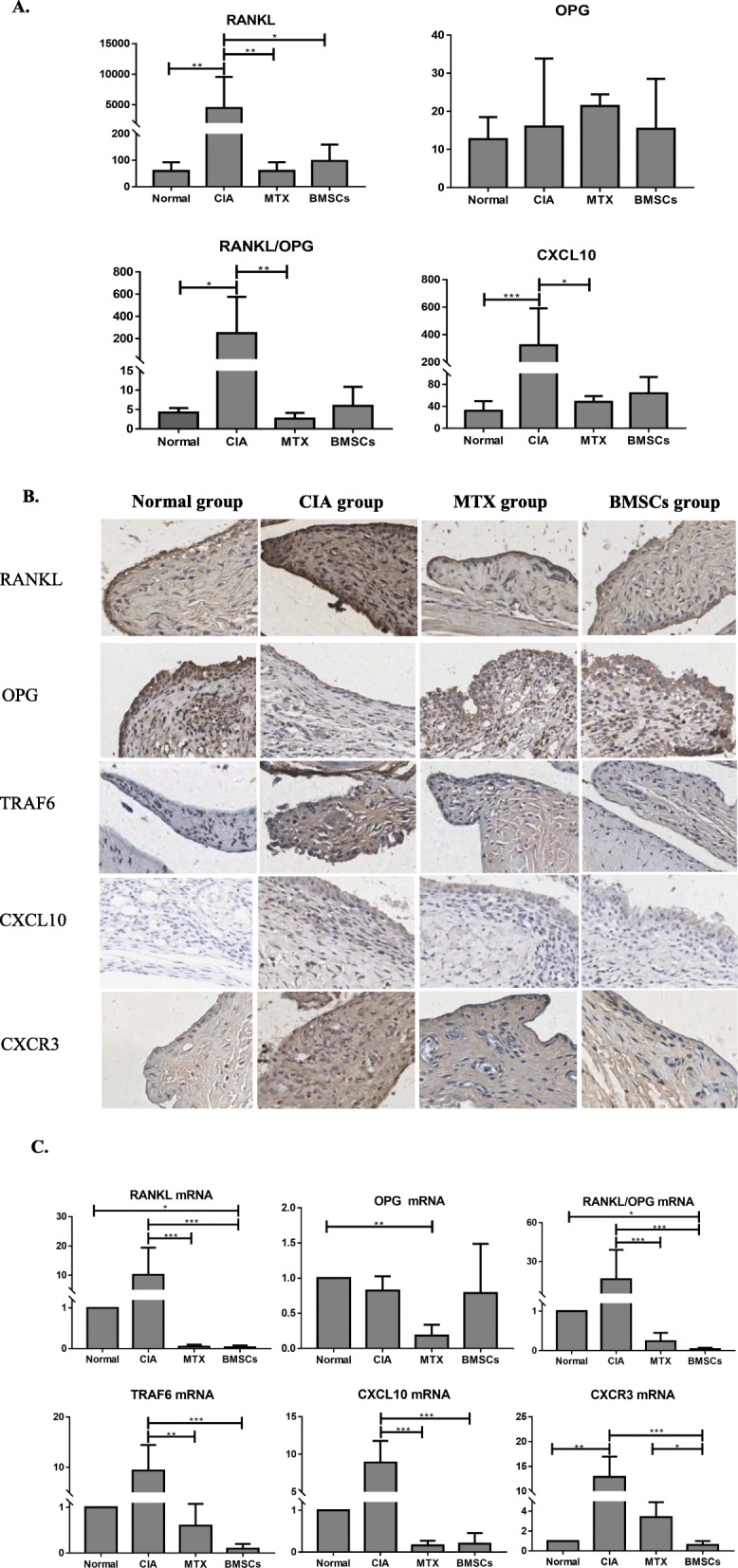
Effects of BMSCs on RANKL/OPG/TRAF6 signaling and the CXCL10/CXCR3 chemokine axis in CIA model rats. **a** Comparison of RANKL, OPG, and CXCL10 levels and RANKL/OPG ratio in the serum among the four groups. **b** RANKL, OPG, TRAF6, CXCL10, and CXCR3 levels in the synovial membrane. **c** RANKL, OPG, TRAF6, CXCL10, and CXCR3 mRNA levels in the synovial membrane. *P < 0.05; **P < 0.01; ***P < 0.001

No significant difference in serum OPG levels was observed among the four groups. However, the differences in RANKL (*P* < 0.001) and CXCL10 (*P* < 0.001) levels and the RANKL/OPG ratio (*P* < 0.01) were significant among the four groups. Compared with those in the normal group, the abovementioned parameters increased in the CIA group. Both BMSC and MTX intervention reduced the serum RANKL levels and the RANKL/OPG ratio in rat serum; however, the difference in the RANKL/OPG ratio between the BMSC and CIA groups was not significant(*P* = 0.073). The difference in the RANKL level among the normal, BMSC, and MTX groups was not statistically significant (*P* = 1). CXCL10 level in the CIA group was higher than that in the normal group (*P* < 0.001). MTX intervention reduced serum CXCL10 levels (*P* = 0.032); however, the difference between the BMSC group and the CIA group was not significant (*P* = 0.535) (Fig. 3a).

### RANKL, OPG, TRAF6, CXCL10, and CXCR3 levels in the synovial membrane

#### The protein levels of RANKL, OPG, TRAF6, CXCL10, and CXCR3 in synovial membrane

Immunohistochemical analysis revealed that cell proliferation increased in the synovial tissue in the CIA group, and synovial cells increased from normal 2–3 layers to 20–30 layers. RANKL, TRAF6, CXCL10, and CXCR3 levels were higher, and OPG levels were lower in the CIA group than in the control group. However, RANKL, TRAF6, CXCL10, and CXCR3 levels in the MTX and BMSC intervention groups were reduced, and OPG levels increased (Fig. 3b).

#### The mRNA levels of RANKL, OPG, TRAF6, CXCL10, and CXCR3 in the synovial membrane

Overall, the relative mRNA expression levels of RANKL (*P* < 0.001), OPG (*P* < 0.01), and TRAF6 (*P* < 0.001) were significantly different among the four groups. The relative mRNA expression of RANKL (*P* = 0.644), TRAF6 (*P* = 0.159), and CXCL-10 (*P* = 0.345) and the ratio of RANKL/OPG mRNA (*P* = 0.512) in the CIA model group were greater than those in the normal group; however, these parameters did not differ significantly among the four groups. Post hoc multiple comparisons revealed that both BMSC and MTX interventions downregulated RANKL (*P* < 0.001, *P* < 0.001) and TRAF6 mRNA (*P* < 0.001, *P* < 0.01), but the difference between the BMSC group and the MTX group was not significant (*P* = 1).

Relative CXCL10 mRNA expression levels were slightly but not significantly higher in the CIA group than in the control group (*P* = 0.345). Both BMSC and MTX interventions significantly downregulated CXCL10 mRNA (*P* < 0.001, *P* < 0.001). However, only the BMSC intervention downregulated CXCR3 mRNA (*P* < 0.001), and there was no significant difference between the MTX and CIA groups (*P* = 0.514) (Fig. 3c).

### Assessment of the migration and colonization of GFP-MSCs and chondrocyte differentiation in the joints of CIA rats

#### Distribution and colonization of GFP-BMSCs in the posterior ankle joints of CIA rats

**Fig. 4:**
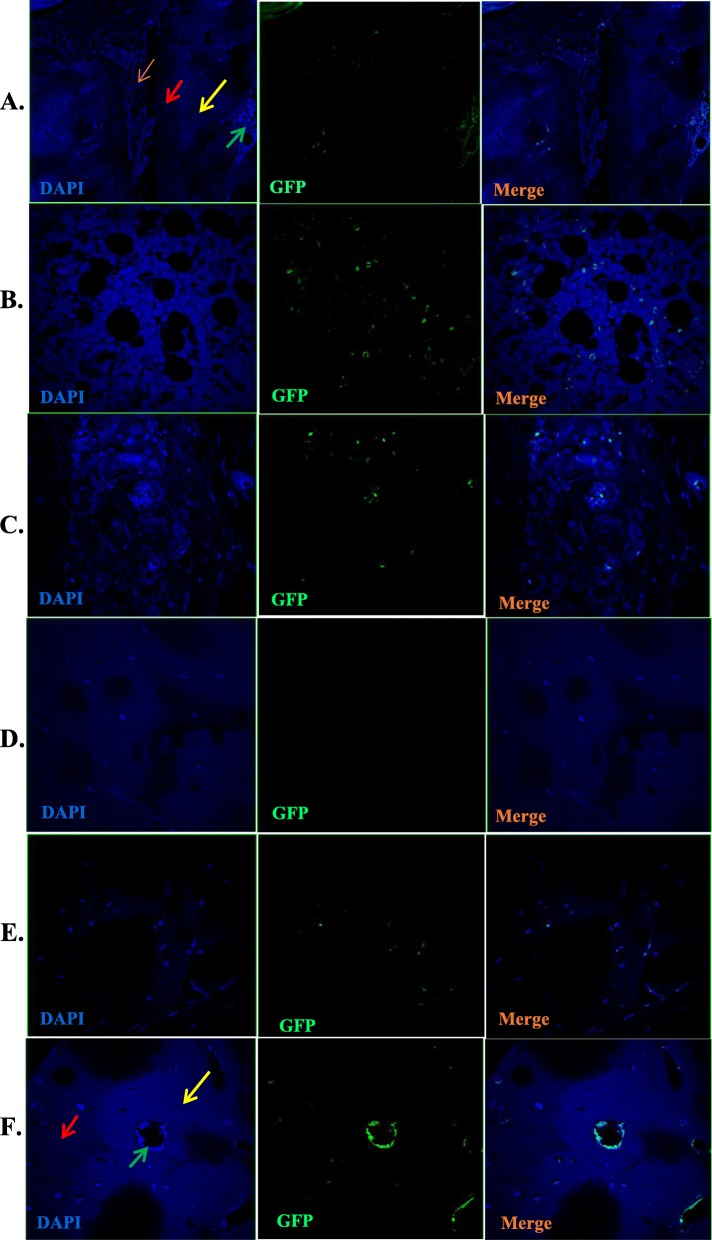
The distribution of GFP-labeled bone marrow mesenchymal stem cells (BMSCs) in the synovial cartilage and bone marrow 3 days after transplantation. **a** BMSCs were distributed in the bone marrow and synovial membrane but not in the cartilage and bone tissue. The yellow arrow denotes bone tissue; orange, synovial tissue; red, cartilage tissue; and green, bone marrow cavity (× 40). **b** GFP+ cells in the bone marrow (× 600). **c** GFP^+^ cells in the synovium (× 600). **d** No GFP+ cells were observed at 3 days in the cartilage and bone tissue (× 600). e After 11 days of transplantation, GFP-positive cells were observed in the articular cartilage, primarily spindle cells (× 600). **f** After 42 days of transplantation, GFP-positive cells were observed in the articular cartilage and bone tissues, primarily comprising oval and spherical cells (× 600). The red arrows denote cartilage tissue; yellow, bone tissues; and green, bone marrow cavities

GFP-MSCs were traced in the posterior ankle joints of CIA model rats on days 3, 11, 30, and 42 after intervention. Three days after transplantation, GFP-positive cells were primarily distributed in the bone marrow and synovial tissue, while no GFP-positive cells were observed in the cartilage tissue (Fig. 4a). GFP-positive cells were observed in the cartilage from day 11 and gradually changed from spindle type to oval/spherical cells (Fig. 4b), and GFP-positive cells were still observed 42 days after transplantation (Fig. 4c).

#### GFP-BMSC differentiation into chondrocytes in vivo

**Fig. 5:**
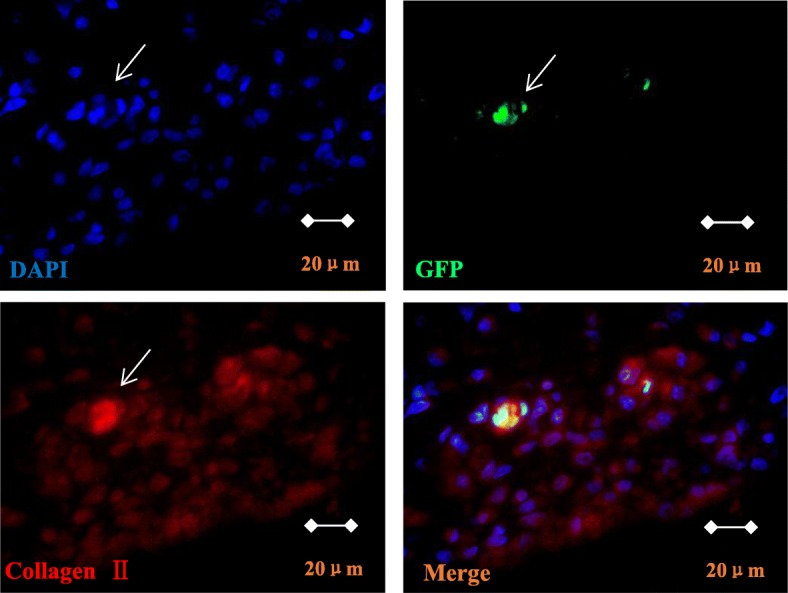
Immunofluorescence staining of bone marrow mesenchymal stem cells undergoing chondrogenic differentiation. GFP/type II collagen double-positive cells (× 1000)

At 42 days after GFP-MSC intervention, GFP/type II collagen double-positive cells were observed at the defect site in the articular cartilage upon immunofluorescence staining (Fig. 5).

## Discussion

Synovial hyperplasia and pannus formation, the primary pathological basis of articular lesions in RA patients, can lead to irreversible cartilage and bone injuries that are difficult to treat.

MSCs are highly proliferative and have immunoregulatory activity. Numerous basic and clinical studies have focused on the use of MSCs for the treatment of RA and other autoimmune diseases and reported that MSCs can inhibit joint inflammation and regulate immune disorders [7, 8]. Furthermore, numerous studies have reported that MSCs can differentiate into adipocytes, osteocytes, vascular endothelial cells, and chondrocytes in vitro with specific inducers. Studies have compared MSCs derived from the bone marrow, umbilical cord, and synovial and adipose tissue and reported that synovial MSCs have the strongest chondrogenic potential, followed by bone marrow and umbilical cord-derived MSCs [9, 10]. Owing to ethical reasons, a normal synovial membrane is not easy to obtain, and it remains controversial whether the long-term inflammatory microenvironment in RA patients affects the differentiation of synovial MSCs. BMSCs are derived from various sources; they are easy to harvest and manipulate, and relatively pure cultures of stem cells can be obtained through various methods. Meanwhile, BMSCs are highly proliferative and active in vitro, and can be extensively amplified in vitro with strong differentiation ability [11], providing an adequate source of raw materials for cell-based therapy. Furthermore, MSC-based interventions can help repair damaged joints in CIA rats. This study shows that MSC transplantation can improve joint symptoms and reduce the AI in CIA, without any adverse reaction. Micro-CT imaging confirmed that osteoporosis and bone destruction in rats were significantly improved, and pathological analysis of the joint revealed that MSCs potentially inhibit synovial hyperplasia and cartilage destruction in CIA rats. Furthermore, clinical control studies have reported that MSC infusion is safe and effective in treating RA patients [12, 13].

However, the underlying mechanism is unclear. BMSCs reportedly survived for 2 weeks after implantation in the coronal pulp chamber of pulpotomized rat maxillary first molars and colonized within the site of potential cytodifferentiation. Furthermore, it was indicated that BMSCs differentiate into cells involved in tissue mineralization in functionally relevant regions [14]. However, the fate of BMSCs upon implantation into RA patients or animal models of RA has remained unknown. Herein, local migration, colonization, differentiation, and the mechanism underlying the inhibition of bone erosion of BMSCs in the joints of CIA model rats were assessed.

MSCs displayed marked anti-inflammatory and cartilage repair functions after transplantation into CIA model rats through the following potential mechanisms:

1. Inhibition of cartilage and bone erosion via paracrine signaling: MSCs modulate the behavior of natural killer cells, dendritic cells, B cells, T cells, osteoclasts, neutrophils, and monocytes/macrophages through numerous molecules including prostaglandin E2 (PGE2), IDO, NO, and IL-10 [15]. In our pre-experiment, we detected various bone destruction-related factors in the serum before and after BMSC intervention and found that BMSCs reduced RANKL and CXCL10 levels. The CXCL10/CXCR3 chemokine axis and RANKL/OPG signaling potentially contribute to joint inflammation and bone destruction during rheumatoid arthritis. Osteoclast formation is primarily regulated by three factors: RANKL, RANK, and OPG [16, 17]. RANKL is a key factor promoting osteoclast differentiation and activation during physiological bone reconstruction and binds to RANK on the surface of osteoclasts to recruit and form a complex with TRAF6, further activating the NF-κB signaling pathway and three pro-mitogen-activated protein kinase pathways (JNK, ERK, and p38) to induce osteoclast formation [16]. OPG is derived from osteoblasts and can inhibit bone resorption by interfering with RANKL/RANK binding [18]. The RANKL/OPG ratio is potentially more relevant than OPG or RANKL level alone in regulating osteoclast formation and bone destruction in RA. A study [19] reported that changes in the RANKL/OPG system, involving an increase in RANKL and reduction in OPG in the peripheral blood and synovial tissue (ST), represent an important mediator of bone resorption in RA-induced osteoporosis. CXCL10 is a 10-kDa protein and is functionally categorized as an inflammatory chemokine. CXCL10 binds to CXCR3 and regulates immune responses by activating and recruiting leukocytes including T cells, eosinophils, monocytes, and NK cells. Serum and/or tissue CXCL10 is reportedly upregulated in RA. CXCL10 and CXCR3 may play important roles in leukocyte homing to inflamed tissues and in the perpetuation of inflammation, thus contributing to tissue damage. In RA, CXCL10 has been detected in the serum, synovial fluid (SF), and ST, and CXCR3 is primarily expressed in ST [20]. Studies on both animal and human RA reported that RANKL induced CXCL10 expression in osteoclast precursors, and CXCL10 in turn upregulated RANKL in CD4^+^ T cells [21]. Thus, the cross-talk between CXCL10 and RANKL, or other cytokines such as TNF-α, may be responsible for the initiation and/or aggravation of inflammation and bone erosion in RA [22]. This study shows that RANKL protein and mRNA levels and RANKL/OPG ratio in serum and ST, and TRAF-6 levels in ST increased in CIA rats and decreased after BMSC intervention. However, BMSC intervention could not alter OPG levels either in the serum or in the synovial membrane. Subsequently, a study on the effect of BMSCs on the CXCL10/CXCR3 chemotaxis axis revealed that serum CXCL10 levels in CIA model rats increased slightly but not significantly in comparison with those in normal rats. However, a pervious study indicated that serum and tissue CXCL10 levels increased in CIA. These inconsistencies in previous reports may be resolved by increasing the sample size. Furthermore, we found that CXCL10 and CXCR3 mRNA and protein in ST were significantly upregulated, consistent with previous reports [20, 23], and upon treatment with BMSCs, they were downregulated. In particular, CXCR3 downregulation was more marked in the BMSC than in MTX intervention groups. Hence, inhibition of related factors of bone destruction, such as RANKL, TRAF-6, CXCL10, and CXCR3, may be a primary mechanism underlying the reduction in cartilage erosion and bone destruction in CIA.
2. Tissue repair: MSCs can differentiate into chondrocytes, osteocytes, and adipocytes in vitro through self-differentiation of damaged cells to replace damaged cells or promote tissue repair. This study also focused on the mechanism underlying BMSC differentiation in vitro and repair of cartilage injuries in vivo. Chondrocytes secrete numerous extracellular matrix components, primarily including mucopolysaccharides and collagen. Collagen type II, representing the majority, and collagen type IX are characteristic markers of chondrocytes [1, 24]. This study shows that BMSCs cultured in induction medium for 14 days could differentiate into chondrocytes by toluidine blue staining and type II collagen immunohistochemical method.

Our previous study reported that MSCs in RA reduced disease activity through transcriptional regulation of genes responsible for T cell proliferation, apoptosis, and differentiation and T lymphocyte-associated inflammatory factors in vivo and vitro [5]. However, after transplantation, the distribution and differentiation of MSCs in vivo and the repair mechanism are unclear. Studies [25, 26] indicated that MSCs inoculated into nano-hMSCs in CIA rats, and other fiber scaffolds in vitro were observed among differentiating chondrocytes and eventually repaired joint injuries. Nonetheless, as RA is a systemic autoimmune disease, the stent implantation is limited to single-joint transplantation, and injection of MSCs in the joint cavity often does not regulate systemic immune disorders or joint destruction. For patients with advanced RA involving multiple joints and patients with extra-articular manifestations (such as vasculitis), local transplantation of MSCs is markedly limited. No study has investigated whether MSCs injected intravenously can migrate and display homing at the damaged joint and carry out tissue repair by differentiating into chondrocytes. In this study, BMSCs labeled by GFP were administered to CIA rats via the tail vein, and their distribution was observed at 3 days, 11 days, 30 days, and 42 days after GFP-MSC treatment. Consequently, GFP-positive cells were observed in the bone marrow and blood vessels of synovial tissue 3 days after injection. From days 11 to 42, GFP-positive cells increased gradually and were observed on cartilage injury engraftment, with changes in the cell morphology from long spindle-shaped cells to oval cells, which was consistent with the surrounding chondrocytes. Systemic infusion of MSCs can play a systemic immunomodulatory role, and chemotaxis to inflammation and damaged joints can locally inhibit joint inflammation and promote repair of joint injuries. The homing mechanism primarily includes the following phenomena: (1) active migration—MSCs express various chemokines and growth factor receptors [27, 28], suggesting that the ligand of soluble receptors may play certain roles in the homing of MSCs. Chemokine receptors (CXCR1, CXCR2, and CXCR4), CC chemokine receptors (CCR1 and CCR2), vascular endothelial growth factor receptor-1 (Flt-1), and platelet-derived growth factor receptors (PDGFR-a and PDGFR-b) and their respective ligand IL-8, stromal cell-derived factor 1 (SDF-1), macrophage inflammatory protein 1a (MIP-1a), monocyte chemokine protein-1 (MCP-1), placental growth factor (PLGF), and platelet-derived growth factor (PDGF) have been extensively investigated in the homing of MSCs; (2) passive intercept—a study [29] has reported that after intravenous transplantation, MSCs are largely trapped in the lungs, liver, and spleen with abundant capillaries. Schrepfer et al. [30] reported that when MSCs were intravenously injected into rats pretreated, the number of MSCs entrapped by pulmonary microvessels decreased. Therefore, homing of MSCs may also be associated with local microvascular changes, increased capillary permeability, hemostasis, and passive retention. This study shows that BMSCs can be chemotactic to inflammatory joints in CIA rats; the underlying mechanisms are potentially associated with the expression of various chemokines and increased vascular permeability in the local inflammatory microenvironment. The specific underlying mechanism needs further study. To investigate whether transplanted MSCs can differentiate at the site of joint injury, 42 days after systemic administration of GFP-MSCs into CIA rats, the posterior ankle joints of the rats were prepared for immunofluorescence staining. Some of the GFP-positive cells contained cartilage-specific type II collagen-labeled cells, confirming that some exogenous BMSCs were transplanted in the cartilage tissue and differentiated into chondrocytes.

Therefore, MSC transplantation can improve joint inflammation and cartilage and bone injury in CIA rats. Transplanted BMSCs can undergo chemotaxis, migration, and colonization at damaged joints and eventually differentiate into chondrocytes to repair the damaged joints.

## Conclusions

BMSCs reduce synovial hyperplasia and cartilage destruction in CIA rats, and their effects may be achieved by inhibiting factors related to bone destruction and repairing damaged cartilage tissue. First, serum CXCL10 and RANKL levels and CXCL10, CXCR3, RANKL, and TRAF-6 levels decreased in synovial tissues of CIA rats in the BMSC group. Furthermore, transplanted MSCs migrated and colonized the site of inflammation and injury in joints and survived for at least 42 days, finally contributing to cartilage repair by differentiating into chondrocytes.

## Data Availability

The datasets used and/or analyzed during the current study are available from the corresponding author on reasonable request.

## Abbreviations

RA: Rheumatoid arthritis
BMSCs: Bone marrow mesenchymal stem cell
CIA: Collagen-induced arthritis
SAARDs: Slow-acting anti-rheumatic drugs
MTX: methotrexate
GFP-MSCs: Green fluorescent protein-labeled MSCs
AI: Arthritis index
H&E: Hematoxylin and eosin
RANKL: Receptor activator for nuclear factor-κ B ligand
OPG: Osteoprotegerin
TRAF6: TNF receptor-associated factor 6
